# Skeletal and dental maxillary morphological characteristics in patients with impacted canines: systematic review and meta-analysis

**DOI:** 10.1093/ejo/cjad050

**Published:** 2023-08-08

**Authors:** Ieva Gudelevičiūtė, Nerija Spaičytė, Dalia Smailienė

**Affiliations:** Faculty of Odontology, Lithuanian University of Health Sciences, J. Lukšos-Daumanto Str. 2, Kaunas, Lithuania; Faculty of Odontology, Lithuanian University of Health Sciences, J. Lukšos-Daumanto Str. 2, Kaunas, Lithuania; Department of Orthodontics, Lithuanian University of Health Sciences, J. Lukšos-Daumanto Str. 6, Kaunas, Lithuania

**Keywords:** occlusal development, impacted teeth, eruption problems, clinical trials, systematic reviews and meta-analyses

## Abstract

**Background:**

There are a few hypotheses for the origin of palatally impacted canines (PIC). Nevertheless, the results of different studies are controversial.

**Objective:**

Considering the evidence available in the literature to determine the skeletal and dentoalveolar dimensions in patients with PIC using cone beam computed tomography (CBCT).

**Search methods:**

This systematic review adhered to the Preferred Reporting Items for Systematic Reviews and Meta-Analysis Statement. The literature search with no publication date restriction in five databases and hand searching was performed until April 2023.

**Data collection and analysis:**

Data assessing the skeletal and dentoalveolar characteristics of subjects with PIC evaluated with CBCT was extracted, and the studies’ quality was evaluated with the Newcastle-Ottawa Scale (NOS). Skeletal and dentoalveolar characteristics of subjects with PIC were compared with non-impacted subjects or non-impacted sides. MedCalc software was used to perform the meta-analysis. Statistical heterogeneity was assessed using the chi-square and I-square tests.

**Results:**

The initial database search identified a total of 1153 studies. After applying the selection criteria, nine articles were included in the systematic review and meta-analysis. According to the NOS, all included articles were graded as “Good” quality. The meta-analysis showed a non-significant difference in measuring dentoalveolar height, alveolar first molar width, and basal lateral width. Controversial results were observed when evaluating both basal and alveolar first premolar widths. A significant difference was found when assessing anterior alveolar crest height and basal maxillary width.

**Conclusions:**

Studies demonstrated the reduction of both dentoalveolar and skeletal maxillary parameters of the patients with PIC. The meta-analysis indicated that PIC correlates to both vertical and transverse skeletal dimensions of the maxilla. However, the results remain controversial. The findings should be interpreted with caution due to different study designs and unbalanced groups in the included studies; therefore, further research is needed for more reliable conclusions.

**Registration:**

This systematic review and meta-analysis were registered in the International Prospective Register of Systematic Reviews (PROSPERO CRD42022362124)

## Introduction

With a frequency ranging from 1% to 2.5%, the maxillary canine is the second most frequently impacted tooth after the third molars [1]. Palatal canine impaction occurs 2–3 times more frequently than labial does [2]. A small maxillary arch and/or a lack of canine room are associated with the pathogenesis of labially impacted canines [3, 4]. Nevertheless, the precise causes of palatal canine impaction are unclear. The guiding hypothesis and the genetic theory are the two competing hypotheses for the origin of palatally impacted canines (PIC) [5, 6].

Tooth eruption is a physiological process that affects the alveolar bone’s normal development, whereas tooth impaction may prevent the alveolar bone’s regional growth [7]. Although it is possible to hypothesize that tooth impaction can result in less masticatory stimulation of the bone [8], investigations on maxillary and alveolar bone dimensions at the affected site are lacking. Nonetheless, it might be that a smaller maxillary width associates with a higher risk of impaction due to limited space in the dental arch.

Several researchers have attempted to determine whether there is a connection between PIC incidence and the skeletal and dental dimensions of the maxilla. McConnell *et al.* [9] linked inadequate anterior maxillary width to palatal canine impaction. According to Mehta *et al.* [10], risk factors for palatal maxillary canine impaction include inadequate intermolar width and increased palatal depth. However, Langberg and Peck [11] reported no significant difference between the PIC and control patients in the maxillary anterior and posterior widths, examining pretreatment dental casts. According to Al-Nimri and Gharaibeh [12], patients with PIC had a noticeably wider upper arch than other patients did.

Cone-beam computed tomography (CBCT) has made it possible to gather precise information on the bone dimensions by displaying three-dimensional pictures of teeth and bone in high resolution. For subjects with PIC, CBCT can be used for localization, evaluation of root resorption [13], alveolar bone density [14], or maxillary skeletal and dental parameters [4, 15–22]. However, the latter varies and seems contradictory in separate studies.

Due to these considerations, this systematic review aimed to gather current information and evaluate the skeletal and dentoalveolar dimensions using CBCT in individuals with PIC.

## Methods

### Protocol and registration

These systematic review and meta-analysis were conducted and reported following the guidelines of Preferred Reporting Items for Systematic Reviews and Meta-Analysis (PRISMA) and was registered in the International Prospective Register of Systematic Reviews (PROSPERO CRD42022362124).

### Focus question

According to the Participants Intervention Comparison Outcome Study design scheme, the study planned to include randomized, prospective, and retrospective controlled trials (S) on human patients of any age, ethnicity, or sex with palatally impacted maxillary permanent canines (P). The intervention (I) was defined as the CBCT of subjects with PIC, and the comparison (C) was made between patients with impacted and normally erupted canines or between impacted and non-impacted sides. The primary outcome (O) evaluated was maxillary skeletal morphological characteristics (basal maxillary width (BMW), height of alveolar crest (AACH), basal first premolar, and basal lateral widths (BLWs)). The secondary outcome was dentoalveolar variables (intermolar and inter-premolar distance, anterior dentoalveolar height (ADH)). The developed focus question was: What are the maxillary structure variations in subjects with palatally impacted maxillary canines?

Five electronic databases (PubMed, Cohrane Library, Sciences Direct, Web of Science, and Springer Link) were searched systematically (Table 1). This was supplemented with a search of the Directory of Open Access Journals, Digital Dissertations, metaRegister of Controlled Trials, and Google Scholar.

**Table 1.** Data source and search strategies.

Database	Keywords
PubMed	((“Tooth,Impacted”[Mesh])AND(“Cuspid”[Mesh]))AND(“Cone-BeamComputedTomography”[Mesh])AND (measurements OR dimensions OR width)
Cohrane Library	((((impacted canine) OR (displaced canines)) AND (cone beam computed tomography)) OR ((impacted canine) OR (displaced canines) AND (CBCT)) AND (dimensions))
Sciences Direct	
Web of Science	
Springer Link	

#### Inclusion criteria

- randomized, prospective, and retrospective studies published in English,
- patients diagnosed with palatal impaction of the maxillary permanent canine, and
- CBCT images with radiological evaluation measurements before treatment.

#### Exclusion criteria

- literature reviews, case reports, and series;
- panoramic or dental radiographs and dental casts used for evaluation; and
- patients with genetic syndromes (craniofacial syndromes, cleft lip, or palate), severe facial malformations or systemic diseases, previous orthodontic treatment, dento-maxillary traumas, or agenesis.

### Selection of studies

Before beginning the search in the selected databases, the three investigators discussed the search strategy. Two researchers then performed the study selection independently. Selection and filtration were done by assessing the titles of the articles and their abstracts; duplicates were removed. If the article met the inclusion criteria, the entire article was read to make the final decision. In addition, the reference/citation lists of the included trials were manually searched for any additional studies. Disagreements were resolved by consensus between the two reviewers and a third author was consulted when necessary. The last search was conducted on 22 April 2023.

### Data extraction

Two authors independently extracted the study characteristics, including design, sample size, patient age and sex, and maxillary morphological characteristics measurements (BMW, alveolar bone height, inter-premolar width, and intermolar width).

### Assessment of methodological quality

The quality of the included study protocols was assessed by investigating full-text articles. The Newcastle-Ottawa Scale (NOS) risk of bias assessment tool was used to evaluate the methodological quality of non-randomized clinical studies [23]. Three domains were considered: (i) selection of study groups (four points) (ii), comparability of groups (two points), and (iii) ascertainment of outcomes (three points). Cases of disagreement were resolved by consultation and discussion with a third party.

### Synthesis of results

A meta-analysis was conducted on the quantitative data using MedCalc v14.8 (MedCalc Software bvba, Ostend, Belgium). Considering the high clinical heterogeneity among the included studies, a random effect model was used for analysis. Statistical heterogeneity was assessed using the chi-square and I-square tests. I2 values of 25%, 50%, and 75% indicated low, moderate, and high heterogeneity, respectively. *P* values ≤0.05 were considered statistically significant. Characteristics included in the meta-analysis were the AACH, basal first premolar width (BPMW), basal lateral width (BLW), ADH, alveolar first molar width (AMW), and alveolar first premolar width (APMW). Studies with methods and outcomes that could not be quantitatively analyzed were described qualitatively.

## Results

### Study selection

**Figure 1.**
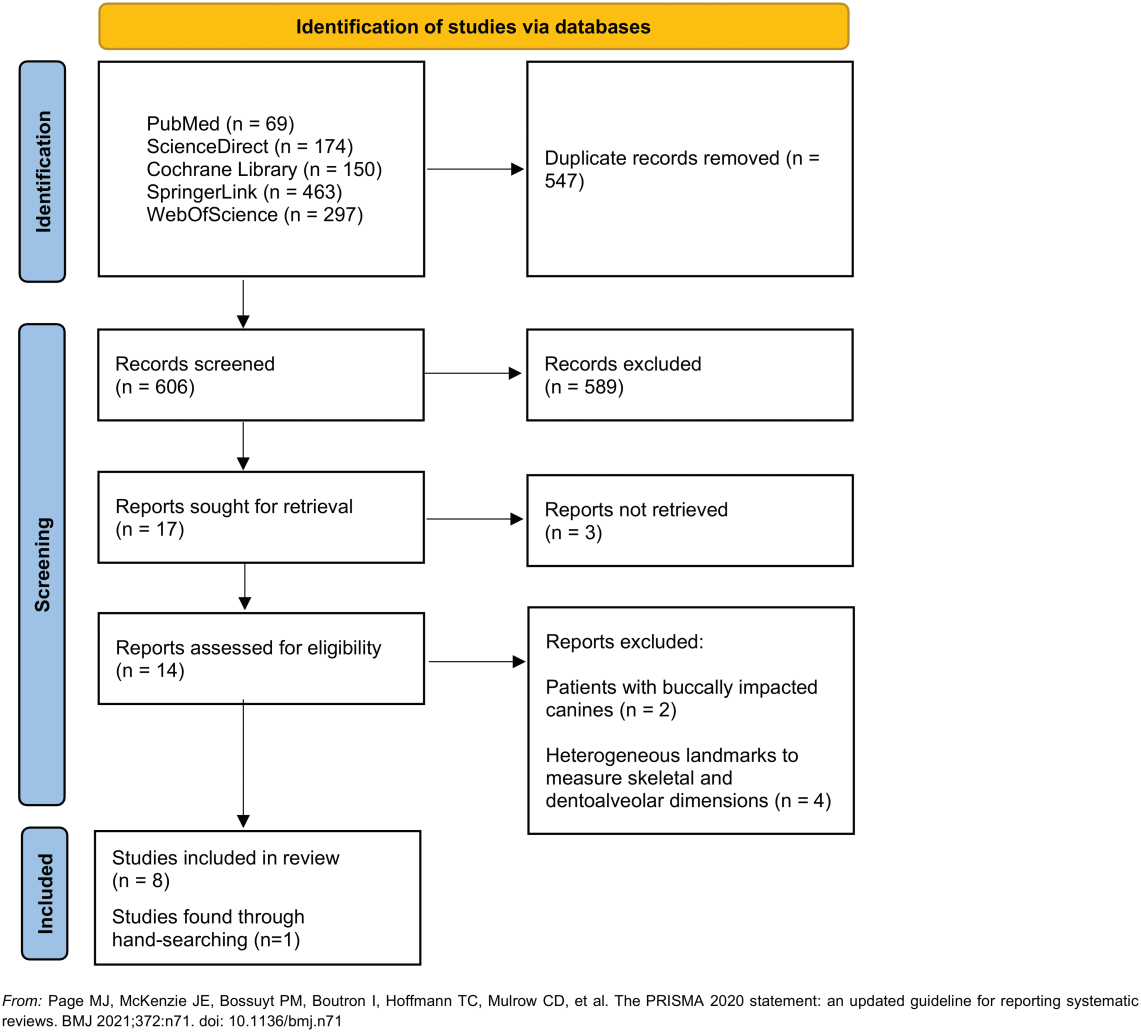
PRISMA selection criteria flow chart.

A total of 1153 articles were identified in the online search engine. Following the removal of duplicates and the review of article titles and abstracts, reports were sought for retrieval and three of them were not retrieved (Supplementary Table S1). Eight articles that met all the selection criteria were chosen. Additionally, one study was found manually. The present review uses nine articles [4, 15–22]. The study selection process is illustrated in a flowchart in Figure 1. In the meta-analysis, nine studies were included [4, 15–22].

### Study characteristics

All selected studies were retrospective with a split-mouth design [15–18] or a control group [4, 18–22]. Seven reviewed articles included only unilateral palatal impactions [15–19, 21, 22] and two included bilateral cases as well [4, 20].

Fundamental data extracted from individual studies are presented in Table 2. The average number of patients per study was approximately 67 (with a minimum of 15 and a maximum of 100 patients). The total number of included patients was 602 with the age ranging from 12 to 60 years.

**Table 2.** Data of interest.

	Author, year	Study design	Sample size (M/F)	Mean age (SD), Range (years)	Groups (*n*)	Eligible outcome
1.	Tadinada *et al.*, 2015 [15]	RS Split-mouth	39 (16/23)	17.07 (−)12–29	SI: impacted side [39] SC: controlside [39]	• Anterior height of alveolar crest (AACH) • Buccolingual width of the alveolar ridge • Arch perimeter
2.	Sar *et al.*, 2022 [16]	RS Split-mouth	30 (13/17)	19.8 (3.3) –	SI: impacted side [15] SC: controlside [15]	• Anterior height of alveolar crest (AACH) • Anterior dentoalveolar height (ADH) • Basal lateral width • Basal first premolar width (BPMW)
3.	D´Oleo-Aracena *et al.*, 2017 [17]	RS Split-mouth	28 (11/17)	M 22.09 (4.70) F 23.12 (5.17) –	SI: impacted side [28] SC: controlside [28]	• Anterior height of alveolar crest (AACH) • Anterior dentoalveolar height (ADH) • Basal lateral width • Basal first premolar width (BPMW)
4.	Montes-Díaz *et al.*, 2022 [18]	RS	100 (G1:21/29;G2:23/27)	32.8 (9.16) 20–45	GI (SI/SC): unilateral PIC [50] GC: control patients [50]	• Anterior height of alveolar crest (AACH) • Basal maxillary width (JL-JR) • Arch length
5.	Genc and Karaman, 2022 [19]	RS	30 (G1: 8/7G2:7/8)	15.82 (1.62) 13–18	GI:unilateralPIC [15] GC: control patients [15]	• Basal maxillary width (ML-MR) • Basal first premolar width (BPMW) • Palatal vault depth • Alveolar first premolar width (APMW) • Alveolar first molar width (AMW)
6.	Yan *et al.*, 2013 [4]	RS	138 **(–)**	– 12–30	GI:unilateral/bilateralPIC (69) GC: matched controls (69)	• Basal maxillary width (JL-JR) • Alveolar first molar width (AMW) • Alveolar first premolar width (APMW)
7.	Hong *et al.*, 2015 [20]	RS	85 (G1: 11/22;G2: 22/44)	18.2 (–) 10–42	GI: unilateral/bilateral PIC [33] GC: matched controls (66)	• Basal maxillary width (ML-MR) • Basal first premolar width (BPMW) • Alveolar first molar width (AMW) • Alveolar first premolar width (APMW)
8.	Arboleda-Ariza *et al.*, 2018 [21]	RS	92(–)	G1 23.1 (10.6) G2 26.5 (6.05)	GI: unilateral PIC [25] GC: control patients (67)	• Basal maxillary width (ML-MR) • Basal first premolar width (BPMW) • Alveolar first molar width (AMW) • Alveolar first premolar width (APMW)
9.	Elhamshary *et al.*, 2023 [22]	RS	60 (–/–)	– (–) 15–60	GI: unilateral PIC [20] GC: control patients [20]	• First premolar width (APMW) • First molar width (AMW) • Basal first molar width (BMW)

RS, retrospective study; GI, impactiongroup; GC, controlgroup; SI, impactedside; SC, controlside; (-), not defined.

All the studies [4, 15–22] received ethical approval from their ethical committee/review board.

### Methodological quality assessment of included studies

**Figure 2.**
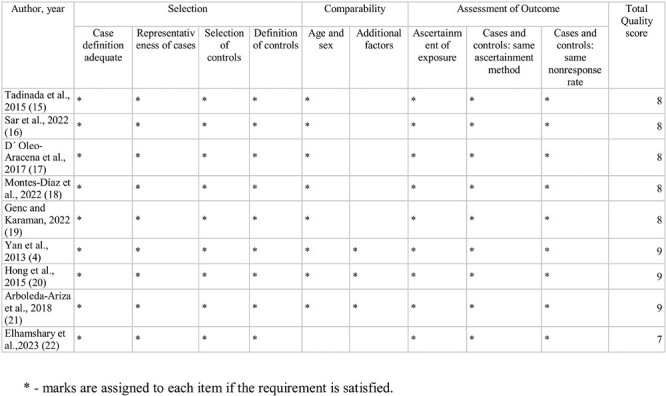
Quality assessment of the included studies according to Newcastle-Ottawa Scale (NOS).

All articles were rated as “Good” quality with a low risk of bias (Figure 2). Primarily, points were lost due to a lack of controls for possible confounders. The NOS scores of all studies ranged from 7 to 9 out of a possible 9 (4, 15–22).

### Results of individual studies

The outcomes of all individual studies for the primary and secondary outcomes are summarized in Tables 3 and 4, one presenting the skeletal and another presenting dentoalveolar measurements.

**Table 3.** Skeletal maxillary morphological characteristics in patients with PIC.

Nr.	Author, Year	Basal maxillry width (BMW) Mean ± SD (mm)				*p* Value	Height of alveolar crest (AACH) Mean ± SD (mm)						Basal first premolar width (BPMW)Mean ± SD(mm)						Basal LateralWidth (BLW)Mean ± SD(mm)		
		ML-MR		JL-JR																	
		GI	GC	GI	GC		GI	GC	*p* Value	SI	SC	*p* Value	GI	GC	pvalue	SI	SC	pvalue	SI	SC	pvalue
1.	Tadinada *et al.*, 2015 [15]									18.12± 2.27	19.25 ± 2.11	0.00 1^*^									
2.	Sar *et al.*, 2022 [16]									16.84± 2.45	17.11 ± 2.72	0.78				18.5 ± 2. 44	16.47 ± 2 .43	0.03^*^	27.47 ± 1 .7	30.89 ± 1 .41	<0.00 1^*^
3.	D´Oleo-Aracena *et al.*, 2017 [17]									21.10± 3.91	20.92 ± 4.00	0.86 1				12.72 ± 2 .25	14.67 ± 2 .00	<0.00 1^*^	19.01 ± 2 .55	19.69 ± 2 .29	0.298
4.	Montes-Día z *et al.*, 2022 [18]			58.39 ± 3.50	59.77 ± 4.14	0.07 5	18.52 ± 3 .47	20.80 ± 2. 274	<0.0 1^*^	15.15 ± 2.98	16.07 ± 3.28	<0.0 1^*^									
5.	Genc and Karaman, 2022 [19]	61.8 ± 2.4	66.7 3 ± 3.84			0.00 1^*^							35.33 ± 2.77	38.8 ± 3	0.00 4^*^						
6.	Yan *et al.*, 2013 [4]			77.42 ± 5.09	77.83 ±3.37	>0.0 5															
7.	Hong *et al.*, 2015 [20]	60.7 9 ± 2.31	61.1 3 ± 2.73			0.34							50.42 ± 4.93	51 ± 4.58	0.59						
8.	Arboleda-Ariza *et al.*, 2018 [21]												35.56 ± 5.3	37.9 ± 5.1	>0.0 5						
9.	Elhamshary *et al.*, 2023 [22]			62.04 ± 3.38	63.63 ±4.60	0.22															

^*^- Statistically significant.

GI, impaction group; GC, control group; SI, impacted side; SC, control side; ML-ML, outer edges of the right and left sides of the maxillary base at the level of first molars along the reference plane on the nasal base level; JL-JR- linear measurement between points on the jugal process at the intersection of the outline of the maxillary tuberosity and the zygomatic buttress.

**Table 4.** Dentoalveolar maxillary morphological characteristics in patients with PIC.

Nr.	Author, year	Alveolar first premolar width (APMW) Mean ± SD (mm)			Alveolar first molar width (AMW) Mean ± SD (mm)			Anterior dentoalveolar height (ADH) Mean ± SD (mm)					
		GI	GC	*P* value	GI	GC	*P* value	CI			LI		
								SI	SC	*P* value	SI	SC	*P* value
1.	Sar *et al.*, 2022 [16]							25.17 ± 3.48	24.68 ± 3.41	0.7	23.63 ± 4.16	23.99 ± 3.21	0.79
2.	D´Oleo-Aracena *et al.*, 2017 [17]							29.72 ± 4.51	29.65 ± 4.46	0.948	27.81 ± 3.68	28.17 ± 3.68	0.713
3.	Genc and Karaman, 2022 [19]	43.53 ± 2.47	48.53 ± 3.46	0.000^*^	52.93 ± 2.55	59.2 ± 2.62	0.000^*^						
4.	Yan *et al.*, 2013 [4]	36.87 ± 2.43	37.10 ± 2.20	>0.05	48.55 ± 2.81	48.90 ± 2.84	>0.05						
5.	Hong *et al.*, 2015 [20]	40.54 ± 3	40.14 ± 1.75	0.51	45.94 ± 2.29	45.67 ± 1.76	0.59						
6.	Arboleda-Ariza *et al.*, 2018 [21]	41.2 ± 4.6	45.2 ± 2.3	0.000^*^	53.58 ± 4.4	57.2 ± 2.7	0.000^*^						
7.	Elhamshary *et al.*, 2023 [22]	43.34 ± 2.48	45.41 ± 3.13	0.023^*^	56.60 ± 2.25	56.58 ± 4.02	0.98						

^*^-Statistically significant.

GI, impaction group; GC, control group; SI, impacted side; SC, control side; CI, a distance from the upper central incisor edge; LI, a distance from the lateral incisor edge.

### Skeletal parameters

#### Alveolar bone measurements

Alveolar bone measurements were reported in four articles [15–18]. All of them evaluated the AACH as a distance from the floor of the nasal fossa to the alveolar crest measured at the level of the canines. The mean alveolar crest height measured toward the impacted canine side ranged from 15.15 ± 2.98 mm to 21.1 ± 3.91 mm, and on the non-impacted side from 16.07 ± 3.28 mm to 20.9 ± 4.00 mm; the differences were statistically significant in two studies [15, 18]. The meta-analysis revealed a significant difference in the anterior height of the alveolar crest between impacted and non-impacted sides [15–18] (Supplementary Figure S1).

Only one article [15] evaluated alveolar bone bucco-palatal (BP) width. At the height of 2 mm above the alveolar crest, there was a statistically significant difference (*P* < 0.0001) between the BP width of the impacted side (6.87 ± 1.08 mm) and the non-impacted side (8.70 ± 1.13 mm). In contrast to the non-impacted side (8.90 ± 1.68 mm), there was no statistical difference in BP width at the height of 6 mm on the impacted side (8.55 ± 2.23 mm) as well as at 10 mm (9.51 ± 2.26 vs. 10.26 ± 2.31).

### Basal lateral width

The BLW was studied in two split-mouth design studies [16, 17]. In both cases, BLW was measured as the distance between the anterior nasal spine and the outermost dentoalveolar rim on the side of the impacted canine and the side without impaction. The mean BLW on the impacted side ranged from 19 ± 2.55 mm to 30.9 ± 1.41 mm and on the non-impacted side from 19.7 ± 2.29 mm to 27.5 ± 1.7 mm. Only one study reported a statistically significant outcome [16]. The meta-analysis showed a non-significant difference in the BLW between impacted and non-impacted sides (Supplementary Figure S1).

### Basal maxillary width

There were five articles published on evaluating the BMW [4, 18–20, 22]. In two [19, 20], BMW was measured from the outer of the maxillary base at the level of first molars along the reference plane on the nasal base level (ML-MR), and the other three [4, 18, 22] measured the JL-JR Ricketts distance (linear measurement between points on the jugal process at the intersection of the outline of the maxillary tuberosity and the zygomatic buttress). Two studies [4, 20] assessed both unilateral and bilateral impactions. Only one research [19] found a statistically significant difference between groups when measuring BMW between jugal points, including only unilateral PIC. In two homogenous studies [18, 22], the mean BMW ranged from 58.4 ± 3.50 mm to 62.04 ± 3.38 mm in patients with PIC, and from 59.8 ± 4.14 mm to 63.63 ± 4.60 mm in patients with normally erupted canines. A meta-analysis of these two studies [18, 22] revealed a significant difference in the BMW (Supplementary Figure S1).

### Basal first premolar width

Five studies [16, 17, 19–21] reported on BPMW: distance from the middle palatine raphe to proximal alveolar bone crest between the canine (deciduous or permanent) and first premolar on each side, measured on the axial cut at bone crest level. The mean BPMW on the impacted sides of two split-mouth studies [16, 17] ranged from 12.7 ± 2.25 mm to 18.5 ± 2.44 mm and from 14.7 ± 2.00 mm to 16.5 ± 2.43 mm on the non-impacted sides. A meta-analysis revealed a non-significant difference in the BPMW between impacted and non-impacted sides [16, 17] (Supplementary Figure S1).

One study [20] evaluating both unilateral and bilateral PIC showed no significant difference. In the remaining two studies [19, 21] the main BPMW ranged from 35.3 ± 2.77 mm to 35.6 ± 5.3 mm in the impacted subjects versus 37.9 ± 5.1 mm to 38.8 ± 3 mm in non-impacted controls, and only the study by Genc and Karaman [19] showed statistically discernible results. However, a meta-analysis of these two studies [19, 21] revealed a significant difference in the BPMW between impacted and non-impacted patient groups (Supplementary Figure S1).

### Dentoalveolar parameters

#### Anterior dentoalveolar height

Two studies reported on ADH [16, 17]. This parameter was characterized as a distance from the upper central (ADH-CI) and lateral (ADH-LI) incisor edges to the floor of the nostrils on the side of the impacted canine and the side without impaction by drawing a straight line parallel to the midsagittal plane. The mean ADH-CI varied from 25.17 ± 3.48 mm to 29.72 ± 4.51 mm on the impacted side and from 24.68 ± 3.41 mm to 29.65 ± 4.46 mm on the non-impacted side. A meta-analysis showed a non-significant difference in the ADH-CI between sides (Supplementary Figure S2). The mean ADH-LI ranged from 23.63 ± 4.16 mm to 27.81 ± 3.6 mm on the impacted side and from 23.99 ± 3.21 mm to 28.17 ± 3.68 mm on the non-impacted side. A non-significant difference in the ADH-LI between sides was found when performing a meta-analysis as well (Supplementary Figure S2).

#### Alveolar first molar width

AMW was estimated in five studies [4, 19–22]. In two studies, it was measured between the deepest points of the central fossae of the first molars, with the mean AMW ranging from 45.94 ± 2.29 mm to 48.55 ± 2.81 mm in patients with unilateral/bilateral PIC and from 45.67 ± 1.76 mm to 48.90 ± 2.84 mm in the control patients. A meta-analysis of these two studies [4, 20] revealed a non-significant difference between groups.

In the other three studies [19, 21, 22] evaluating only unilateral PIC, the AMW dimension was measured between the most occlusal points of the maxillary alveolar process on the first molar coronal slice. The range of this measurement in the impaction group was from 52.93 ± 2.55 to 56.60 ± 2.25 mm and in the control group, from 56.58 ± 4.02 mm to 59.2 ± 2.62 mm. Two of these studies [19, 21] showed measurements that were statistically significant. However, a meta-analysis showed a non-significant difference (Supplementary Figure S2).

#### Alveolar first premolar width

Five studies [4, 19–22] evaluated APMW. Two studies assessed patients with unilateral/bilateral PIC [4, 20] measured in between the deepest points of the central fossae of the maxillary first premolars, the other three [19, 21, 22] assessed only unilateral PIC—between the most occlusal points of the maxillary alveolar process on the first premolar coronal slice. In all but one study [20], a decrease in first premolar width in the PIC group compared to the control group was found. Genc and Karaman [19] reported statistically significant results with a decrease in the first premolar width of 5 mm. A meta-analysis of the three studies [19, 21, 22] revealed a significant difference in the APMW between unilateral PIC and non-impacted patient groups (Supplementary Figure S2). However, a non-significant difference was observed when assessing APMW in a meta-analysis of studies done by Yan *et al.* [4] and Hong *et al.* [20] where unilateral and bilateral PIC patients were included (Supplementary Figure S2).

#### Arch length

Arch length (AL) was assessed in two studies [15, 18]. One measured it from the distal surface of the first molar to the intermaxillary suture and compared the impacted side to the non-impacted side. The results were 41.74 mm and 43.54 mm, respectively [15]. Another study [18] evaluated AL between patients with PIC and without, measuring it from the mesial surface of the first permanent molar on one side to the mesial surface of the first permanent molar on the contralateral side. The study found that in the PIC group, there was an average of 1.68 mm less space available with respect to the control group (69.37 ± 3.83 mm vs. 71.05 ± 3.25 mm) [18]. In both studies, the measurements were statistically significant.

## Discussion

Maxillary-impacted canines have frequently been a subject of study, along with the associated dentoalveolar and maxillofacial structures. Some authors have suggested a connection between transverse maxillary width and impaction [9, 10, 12], whereas other studies have found no association [11]. This review aimed to analyze quantitative data on maxillary basal and dentoalveolar dimensions in patients with PIC.

The preferred diagnostic technique for identifying impacted teeth is CBCT because it eliminates many of the frequent issues with panoramic radiography, such as superimpositions, overlapping structures, and image blurring [4, 15, 24].

It is reasonable to presume that the canine tooth’s impaction results in altered alveolar dimensions on the impacted side because the alveolar process develops in response to tooth eruption [25, 26]. When analyzing skeletal dimensions, all studies assessing the correlation between BMW and PIC [4, 18–20, 22] reported reduced BMW in the PIC group in comparison with the control group; however, only one [19] research found a statistically significant difference between groups. Due to the high heterogeneity of three [4, 19, 20] studies, a meta-analysis was performed only on two [18, 22]. The results showed a significantly narrower BMW in patients with PIC in comparison with the non-impaction patients.

The alveolar transverse difference at the premolar level (BPMW) was measured in five studies. All except one [16] found narrower maxillae in the impaction group. Two pairs of studies were homogeneous; therefore, a meta-analysis was performed for the studies of Genc and Karaman [19] together with Arboleda-Ariza *et al.* [21], and Sar *et al.* [16] with D´Oleo-Aracena *et al.* [17]. Controversial results between these two meta-analyses were found: one showed no correlation between impacted and non-impacted sides while evaluating BPMW [16, 17], while another revealed a statistically significant difference between impacted and non-impacted patient groups [19, 21]. It appears that PIC patients have generally narrower maxillae at the premolar area compared to non-impaction patients. However, when comparing the impaction and non-impaction sides of the patients with unilateral PIC, the results tended not to differ.

The reason for the aforementioned disagreement could be the different gender distribution in studies: Arboleda-Ariza *et al.* [21] sampled 27% more females than males, whereas in the other three studies [16, 17, 19], the distribution was quite equal. In Arboleda-Ariza *et al.*’s [21] investigation, sex was found to be the sole factor that affected all transverse measurements, and the transverse measurement values of female patients were lower than those of male patients. Additionally, the methodologies of studies (split-mouth studies [16, 17] versus studies including experimental and control groups [19, 21]) could influence different results in the meta-analyses.

When analyzing dental arch dimensions, some researchers included in this review reported reductions in maxillary dentoalveolar width at two levels (AMW and APMW) in the PIC patient groups when compared to controls [4, 19, 21]. However, a meta-analysis revealed significant differences only in the APMW between unilateral PIC and non-impacted patient groups. These results correlated with the relevant basal dimensions that were reduced on the impacted patients as well [19, 21]. This implies that smaller maxillary basal widths are associated with a higher risk of impaction due to limited space in the dental arch. The maxillary width deficit can be diagnosed at a young age, and interceptive treatment can be performed to prevent this issue [27]. The appropriate timing of the maxillary arch expansion procedure could resolve the transverse deficiency and lower the risk of canine impaction [28]. In contrast, Saade *et al.* [29] found wider first molar and premolar widths in patients with palatally displaced canines. This might be due to the different methodologies applied.

Concerning height measurements, a meta-analysis revealed a statistically significant difference between the impacted and non-impacted groups when evaluating skeletal AACH. Thus, it can be assumed that PIC relates to a vertical deficit of the maxilla.

Tadinada *et al.* [15] discovered the impacted side had significantly less BP width than the non-impacted side. In their investigation, the BP width decreased at a level of 2 mm above the alveolar crest but not at 6 or 10 mm apically. The main cause for this was the existence of an impacted canine higher on the alveolar crest. They asserted that the horizontal dimension of bone loss was higher than the vertical dimension. The results of this review, which demonstrate that there were significant variations in width measures (BLW and BPMW), but not in dentoalveolar heights, support this. Lack of alveolar BP width may relate to gingival recession occurrence risk at the end of PIC treatment.

The results of the dentoalveolar parameter evaluation used in this review can be compared to the results of studies with similar methodologies but performed on dental casts. Vitria *et al.* [30] revealed higher levels of maxillary transverse discrepancies in individuals with impacted canines in a sample of patients aged 10–25. In contrast, Ghaffar *et al.* [31] did not find a statistically significant association between maxillary transverse dimensions and potential impaction in patients with mixed dentition. Langberg and Peck [11] did not discover a difference in dental arch widths between palatal impaction and non-impacted canine groups. They emphasized the hypothesis that hereditary variables might have a greater effect on palatal impaction than maxillary transverse deficiency does.

According to Verma and Saravana Dinesh [32], impaction may be caused by the female molar and premolar basal alveolar widths, which are less than those of males. Similar results were noticed in our review as well. These findings are consistent with studies that indicated women were more likely than men to have impacted canines [33, 34].

There is a lack of evidence concerning transverse deficit in the molar area, both skeletal and dentoalveolar. In the premolars area, a meta-analysis showed constriction of both skeletal and dentoalveolar areas comparing PIC patients and controls. Researchers evaluating PIC patients via cephalometry or dental casts also confirmed that PIC is often diagnosed in patients with an absence of noticeable malocclusion. Amini *et al.*’s [35] study showed that PIC occurred more frequently in dental than in skeletal malocclusion cases. According to the Mercuri *et al.*’s [36] findings, skeletal characteristics in PIC patients are mostly normal, with Class I skeletal relationships and lower ranks of Class II and Class III sagittal skeletal features. Additionally, PIC patients had normal values for the Steiner vertical skeletal relationship angles, or rather, they were more likely to have normal facial divergence [36]. Numerous studies have associated the vertical skeletal relationship with PIC, and their findings have linked PIC to a deep bite [36–39]. The frequent absence of malocclusion in PIC patients explains the delayed identification and diagnosis of this problem, which does not allow for the use of preventive therapies [36].

This systematic review provides clinically significant information regarding the morphological characteristics of the maxilla in patients with PIC. The findings imply that transverse dimension should be corrected with more focus, especially at the level of the first premolar.

The review included studies that used different study designs, such as split-mouth or two groups of subjects (experimental and control), and they included growing and non-growing patients. However, none of the articles calculated a reliable sample size, the gender distribution varied among the studies, and ethnicity-specific differences were likely. Due to the high heterogeneity of the studies, all but three meta-analyses were conducted on the results of two studies. Even though it is a sufficient number to perform a meta-analysis [40], additional studies should be included for more reliable results. For these reasons, this systematic review and meta-analysis only reflect the general tendency toward skeletal and dental maxillary characteristics in patients with PIC. Further high-quality studies with larger samples are required.

## Conclusions

The included studies demonstrated the reduction of both dentoalveolar and skeletal maxillary parameters in patients with PIC. The performed meta-analysis indicated that vertical and transverse skeletal dimensions of the maxilla were associated with PIC. Regarding dentoalveolar characteristics, only the difference in first premolar width between groups was statistically significant in the meta-analysis. However, the results remain controversial, and further research is needed for more reliable conclusions.

## Supplementary Material

cjad050_suppl_Supplementary_Figure_S1

cjad050_suppl_Supplementary_Figure_S2

cjad050_suppl_Supplementary_Table_S1

## Data Availability

The authors confirm that the data supporting the findings of this study are available within the article or its supplementary materials.

## Disclaimer

We declare that this manuscript is original, has not been published before, and is not currently being considered for publication elsewhere. We confirm that the manuscript has been read and approved by all named authors and that there are no other persons who satisfied the criteria for authorship but are not listed. We further confirm that the order of authors listed in the manuscript has been approved by all of us. We understand that the corresponding author is the sole contact for the editorial process. He/she is responsible for communicating with the other authors about progress, submissions of revisions, and final approval of proofs.
