# Supplementary figures and images for: Skeletal and dental maxillary morphological characteristics in patients with impacted canines: systematic review and meta-analysis
Source: Eur J Orthod. 2023 Aug 8;45(6):832–41. doi: 10.1093/ejo/cjad050 (PMC10687515; doi:10.1093/ejo/cjad050)

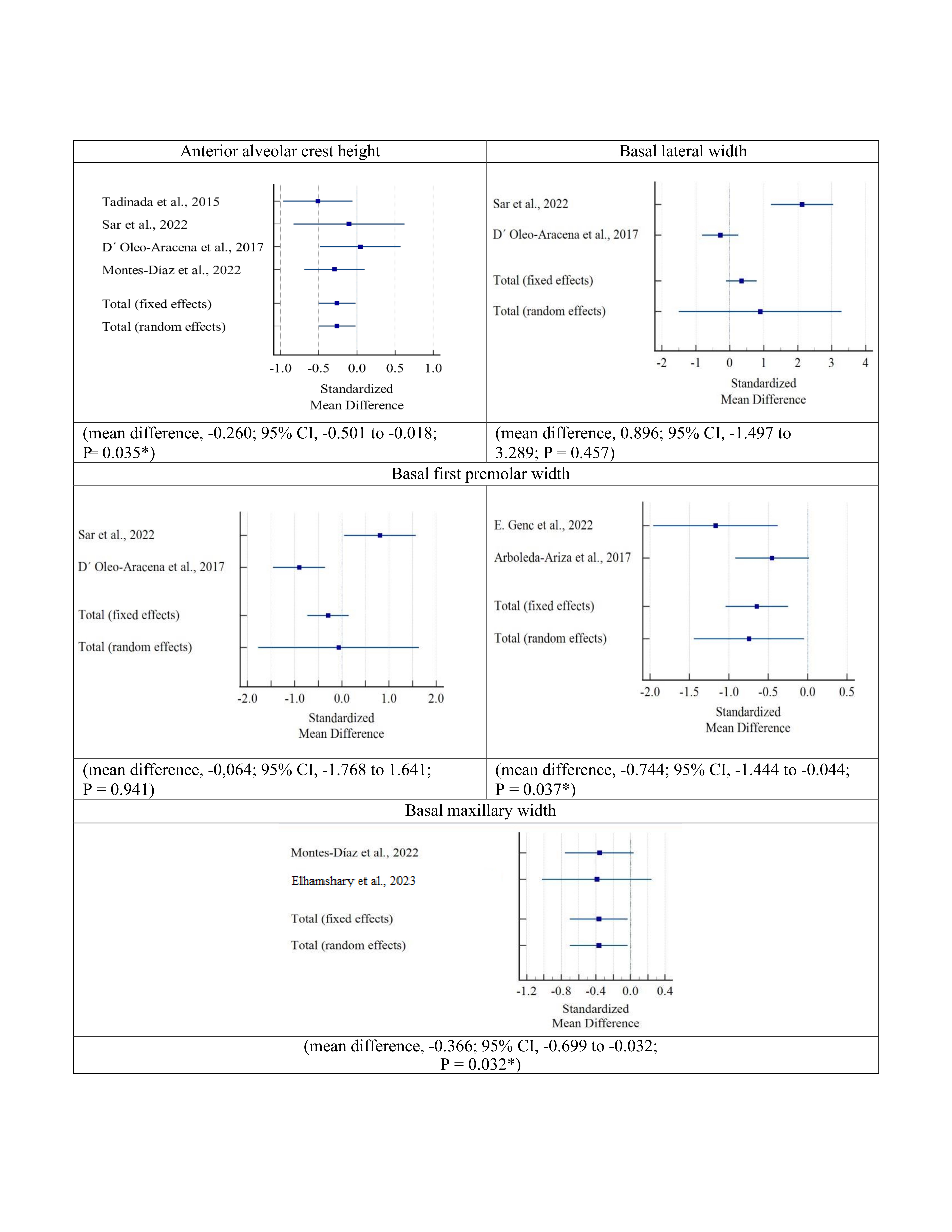

Supplement: cjad050_suppl_Supplementary_Figure_S1 [file cjad050_suppl_supplementary_figure_s1.jpeg]

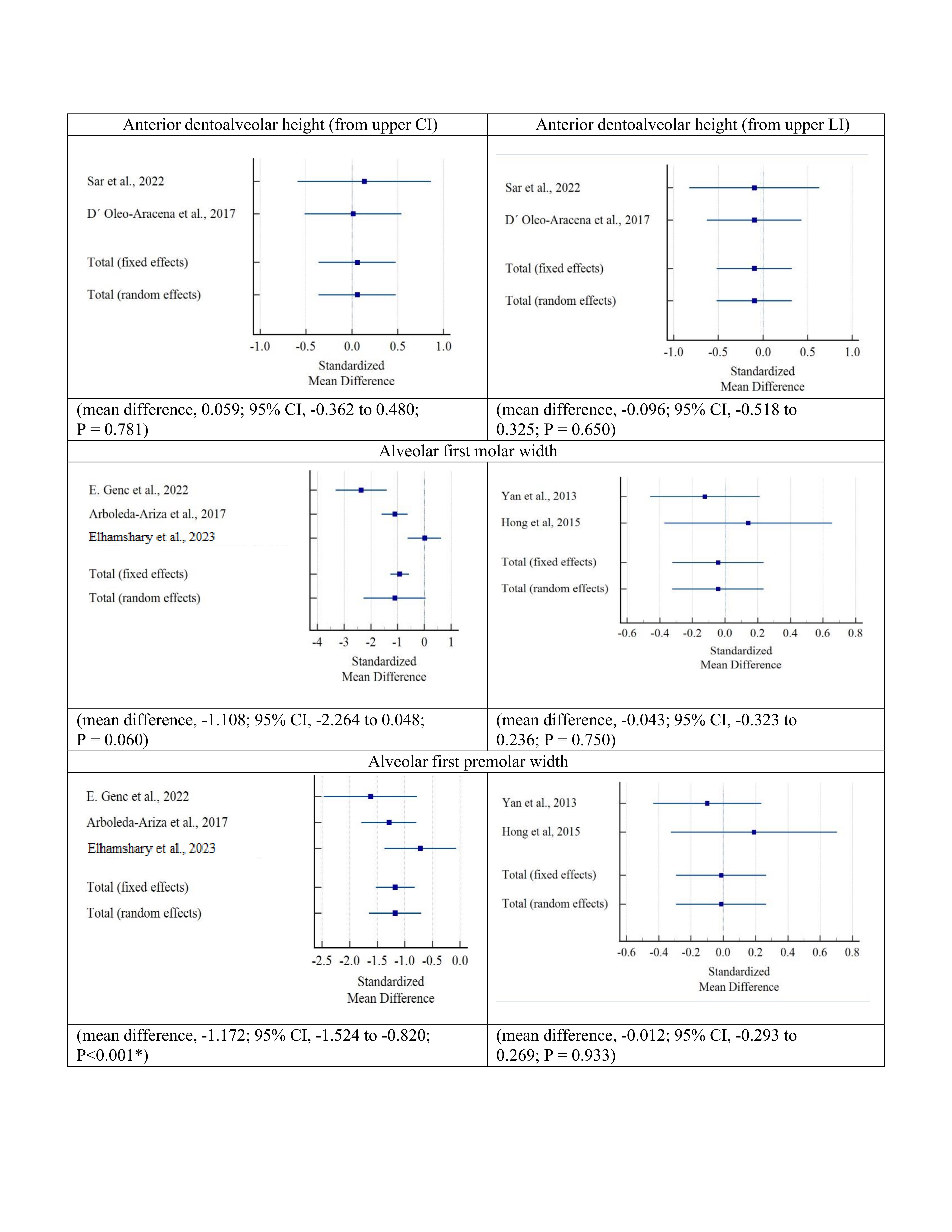

Supplement: cjad050_suppl_Supplementary_Figure_S2 [file cjad050_suppl_supplementary_figure_s2.jpeg]
