# Supplementary material for: Skeletal and dental maxillary morphological characteristics in patients with impacted canines: systematic review and meta-analysis
Source: Eur J Orthod. 2023 Aug 8;45(6):832–41. doi: 10.1093/ejo/cjad050 (PMC10687515; doi:10.1093/ejo/cjad050)
Supplement: cjad050_suppl_Supplementary_Table_S1 [file cjad050_suppl_supplementary_table_s1.docx]

|  | Publication | Reason for not retrieval |
| --- | --- | --- |
| 1. | Miresmaeili A et al. (2019). Morphology of maxilla in patients with palatally displaced canines. International Orthodontics, 17(1), 130–135. | not able to access |
| 2. | Liu AQ, Huang JL, Qian YF (2018). A study on effects of unilateral maxillary canine impaction on the dento-maxillofacial three-dimensional structure. Shanghai kou qiang yi xue. Shanghai journal of stomatology, 27, 79-84 | not able to access |
| 3. | Karacin G, Şenişik NE, Yildirim Derya (2021). Comparison of Lateral Tooth Volume and Morphology Between Buccally and Palatally Localised Unilateral Impacted Maxillary Canine Cases on Cone-Beam Computed Tomography. Journal of Craniofacial Surgery, 32(2), 752-756 | not able to access |
